# Revisiting the dual pathway hypothesis of Chorismate production in plants

**DOI:** 10.1093/hr/uhac052

**Published:** 2022-03-14

**Authors:** Joseph H Lynch

**Affiliations:** Division of Plant and Soil Sciences, West Virginia University, Morgantown, WV 26506, USA

## Abstract

The shikimate pathway, the seven enzymatic steps that synthesize chorismate from phosphoenolpyruvate and erythrose 4-phosphate, produces the last common precursor of the three aromatic amino acids. It is firmly established that all seven enzymes are present in plastids, and it is generally accepted that this organelle is likely the sole location for production of chorismate in plants. However, recently a growing body of evidence has provided support for a previous proposal that at least portions of the pathway are duplicated in the cytosol, referred to as the Dual Pathway Hypothesis. Here I revisit this obscure hypothesis by reviewing the findings that provided the original basis for its formulation as well as more recent results that provide fresh support for a possible extra-plastidial shikimate pathway duplication. Similarities between this possible intercompartmental metabolic redundancy and that of terpenoid metabolism are used to discuss potential advantages of pathway duplication, and the translational implications of the Dual Pathway Hypothesis for metabolic engineering are noted.

## Introduction

The shikimate pathway is ubiquitous across all plants, being necessary for the production of thousands of primary and specialized metabolites [1]. Compounds derived from the shikimate pathway include the aromatic amino acids (AAAs, phenylalanine, tyrosine, and tryptophan), electron transport carriers (ubiquinone, phylloquinone, plastoquinone), pigments (anthocyanins, betalains), hormones (salicylic acid, auxin), defense compounds (glucosinolates, pytoalexins, tannins, benzoxazinoids), antioxidants (flavonoids), signaling compounds (benzenoid and phenylpropanoid volatiles), and structural components (lignin, suberin). Indeed, up to 30% of all photosynthetically-fixed carbon has been estimated to flow through the shikimate pathway [1]. In addition to the importance of this pathway for plant function, survival, and fitness, it has garnered special attention for potential uses of many of the derived compounds due to their agriculturally-, nutraceutically-, pharmacologically-, and industrially-relevant properties, and for its utility as an herbicide target. As such, the plant shikimate pathway has been the subject of intensive study over several decades, and this research has been the subject of multiple comprehensive reviews [1–3].

Research and reviews to date have highlighted the genes and the encoded proteins responsible for the seven enzymatic steps of the shikimate pathway. All seven steps, which are identical to those observed in bacteria and fungi, have been unambiguously shown to function within the plastids [1]. Likewise, the downstream branching pathways that lead to the aromatic amino acids have similarly been characterized with a plastidial localization [4]. However, prior to identification of the genes involved in the shikimate pathway, limited biochemical evidence was reported that was consistent with duplication of at least some steps of the shikimate and subsequent aromatic amino acid pathways in the cytosol, ultimately leading Jensen and colleagues to posit what they termed the “Dual Pathway Hypothesis” [5, 6]. This hypothesis, which suggested the presence of both plastidial and extra-plastidial shikimate pathways operating in parallel, was never widely accepted and gradually fell into obscurity as the dominance, and perceived exclusivity, of the plastidial pathway became apparent [7].

Recently, new evidence has lent fresh support to the presence of some, if not all, steps of the shikimate pathway and subsequent aromatic amino acid biosynthesis outside the plastids in at least some plant species, calling for a renewed look at the Dual Pathway Hypothesis in the genomic age. Here I review the original experimental basis of this hypothesis as it was first proposed, the recent findings that have prompted a revisit to the hypothesis,and the biological and practical implications of the potential complete or partial pathway duplication.

## Classic evidence for the dual pathway hypothesis

**Figure 1:**
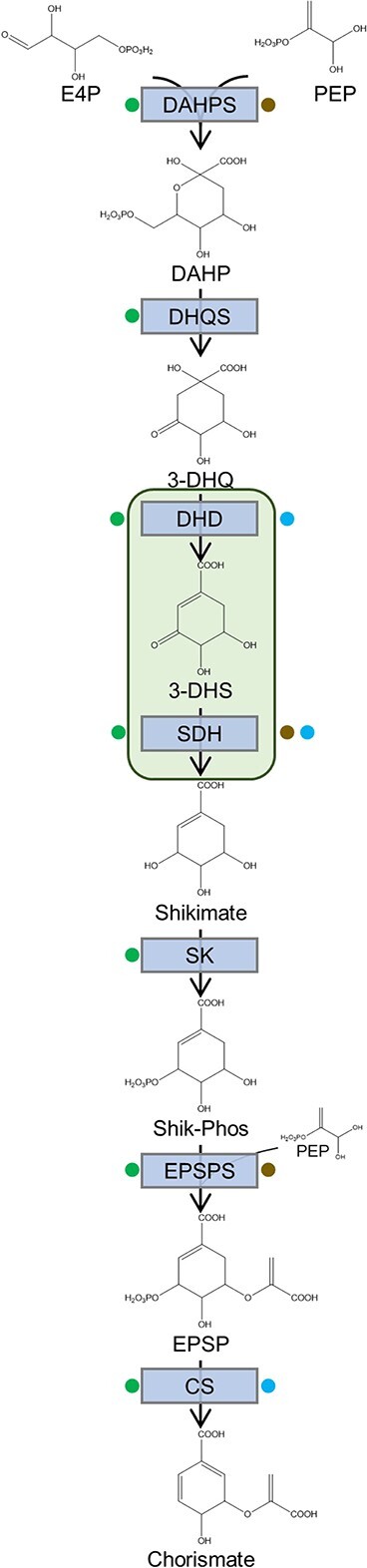
The shikimate pathway. The enzymatic steps of the shikimate pathway, as characterized in microbes in plant plastids, are shown blue boxes. Green shading represents the two steps that are carried out by a single enzyme in plants. Green dots designate enzymes known to be present in plant plastid. Brown and blue dots designate steps for which biochemical or genetic evidence, respectively, is consistent with a cytosolic enzyme in one or more plant species, as described in text. Abbreviations: 3-DHQ, 3-dehydroquinate; 3-DHS, 3-dehydroshikimate; CS, chorismate synthase; E4P, erythrose-4-phosphate; DAHP, 3-deoxy-d-arabino-heptulosonate 7-phosphate; DAHPS, DAHP synthase; DHD, dehydroquinate dehydratase; DHQS, dehydroquinate synthase; PEP, phosphoenolpyruvate; SDH, shikimate dehydrogenase; Shik-Phos, shikimate-3-phosphate; SK, shikimate kinase.

Although the namesake intermediate of the shikimate pathway was first isolated from, and named after, the plant shikimi (Japanese star anise, *Illicium religiosum*) [8], much of the pathfinding work on elucidating the pathway was performed in microorganisms [9]. After characterization of the seven shikimate pathway enzymes in bacteria (Fig. 1), attempts were made to detect the same activities in plant extracts with great success. Subcellular fractionation, and later characterization of encoding genes, repeatedly found these in association with chloroplasts [7, 10], which was consistent with findings from feeding studies demonstrating that isolated chloroplasts were capable of incorporating exogenously provided carbon dioxide and shikimate into the aromatic amino acids [11, 12]. However, as described below, some of these activities were also attributed to extraplastidial enzymes (Fig. 1), forming the basis of the Dual Pathway Hypothesis.

The most thoroughly studied of the extraplastidial shikimate pathway enzymes is that of the DAHP synthase (DAHPS). Using *Vigna radiata*, Rubin et al first reported the presence of two distinct isoforms of DAHPS [13] that were subsequently shown to differ not only in their apparent localization [14], but also in their catalytic and chromatographic properties [14, 15]. The plastidial isoform was stimulated by the presence of Mn^2+^, and was thus designated DS-Mn, while the other was found to be apparently strictly dependent on Co^2+^, and was thus designated DS-Co, although this latter enzyme was also found to maintain high activity with Mg^2+^ in place of the Co^2+^. Subsequent work established that DS-Mn requires a reducing environment and a low pH, whereas Ds-Co maintains activity in absence of reducing agents and prefers an alkaline pH. Furthermore, DS-Co is larger than DS-Mn, with an apparent native molecular weight determined by gel filtration to be around 400 kDa [16], compared to 110 kDa for DS-Mn [17]. The latter is now known to be a multimer of 50 kDa subunits [17, 18], while the former is suspected to be a tetramer of a 115 kDa peptide observed by SDS-PAGE after activity-guided purification from carrot cell suspension culture [19].

Isolation of the gene encoding Ds-Mn from potato [20] and later Arabidopsis [21] demonstrated that it was a Type II DAHPS [22] and enabled the subsequent determination that it is apparently universally retained across all plant species with a clear N-terminal plastidial targeting peptide, and that multiple isoforms are typically present in any given plant genome [1]. The encoding gene for DS-Co remains to be identified. However, the catalytic differences between Ds-Co and Ds-Mn have enabled development of isoform-specific assays, thereby facilitating their further study. Ds-Co is apparently widely distributed throughout the plant kingdom, having been detected from extracts of not just mung bean, but also parsley, potato, carrot, soybean, alfalfa, peas, squash, wheat, rye, *Nicotiana silvestris*, multiple brassica species, and the algae *Chlorella sorokiniana* [14,16,19,23,24]. The physiological role for DS-Co remains unknown, and indeed, its dramatic substrate promiscuity calls into question whether its *in vitro* DAHPS activity is relevant *in vivo* [16]. However, its abundance in *Vitis* cell suspension cultures correlated with production of Phe-derived anthocyanins [25], consistent with a role in providing phenylpropanoid precursors, and its purported substrates PEP and E4P are readily available in the cytosol.

The Dual Pathway hypothesis was also supported by the widespread occurrence of differentially localized isoforms of chorismate mutase (CM), which converts the product of the shikimate pathway, chorismate, to prephenate *en route* to synthesis of Tyr and Phe [26]. As with DAHPS, two isoforms were readily distinguishable by their enzymatic properties, as one (CM-1) was sensitive to feedback inhibition by Tyr and Phe, while the second (CM-2) was apparently devoid of such regulation [27], though was reported to be sensitive to caffeate instead [28]. Cloning of the encoding genes from Arabidopsis later confirmed the existence of three isoforms as distinct enzymes [29, 30], and that one isoform (CM-2) did lack a plastidial transit peptide [30], though the authors were reluctant to conclude that CM-2’s physiological function was indeed related to aromatic amino acid biosynthesis. Nonetheless, the potential for CM-2 to act *in vivo* to synthesize prephenate for the production of Tyr or Phe was further demonstrated through the indirect confirmation that a pool of chorismate resides in the cytosol [31].

A similar, though less well-studied, situation was reported for anthranilate synthase (AS), which also consumes chorismate, but for the synthesis of anthranilate *en route* to synthesis of Trp. As with CM, two isoforms of AS were distinguishable by their localization and sensitivity to an inhibitor, in this case Trp, with only the feedback-sensitive isoform being present in the plastids and the insensitive isoform in the cytosol [32]. Interestingly, treating cells with the Trp analog 5-methyl-tryptophan caused an increase in only the Trp-resistant activity of those cells, suggesting differential transcriptional regulation [32].

Although extra-plastidial activities for other enzymes of shikimate pathway and downstream AAA synthesizing enzymes were sporadically reported, the results were less clear. Whereas DS-Co and CM-2 had distinct biochemical properties to facilitate distinguishing them from their plastidial counterparts, no such differentiation was apparent for other enzymes of the network, and the mere presence of an enzymatic activity in a subcellular preparation may be an artifact of contamination by other cellular components. A chromatographically distinct EPSP synthase (EPSPS) isoform in *Pisum sativum* was reported as present in total shoot extracts, but absent in chloroplast preparations, suggesting a cytosolic localization [10]. However, this is consistent with activity of the plastidial enzyme after translation in the cytosol, but prior to import into the plastids and subsequent transit peptide cleavage, and thus was concluded to be unlikely to be functioning in cytosolic metabolism [33]. In the case of shikimate dehydrogenase (SDH), which similar to EPSP synthase has a chromatographically distinct isoform that is absent in plastids [10, 34], there was some evidence of differential expression. Specifically, although total cellular SDH activity increased over plant development, this increase was not observed in isolated plastids, which was attributed to the presence of a cytosolic isoform that is differentially developmentally regulated compared to the plastidial isoform [35].

## New evidence from the modern age

The first modern challenge to consensus view of the shikimate pathway being exclusively plastidial came with the discovery that cytosolic shikimate is necessary for efficient lignin synthesis. Characterization of the cytosolic enzyme responsible for hydroxylation of the 3-position of the phenyl ring *en route* to production of coniferyl and sinapyl alcohols revealed low activity with free coumaric acid as the substrate, or with the glucose and CoA-esters, yet high activity with the shikimate and quinate esters [36]. The enzyme, later shown to be cytosolic, that catalyzes the formation of these latter esters (hydroxycinnamoyl-CoA:shikimate hydroxycinnamoyl transferase, HCT) was purified from tobacco and its cDNA derived, allowing the determination that the enzyme has a preference for shikimate over quinate [37]. A related branch of the phenylpropanoid metabolic network was discovered with the isolation of a caffeoyl shikimate esterase, shown to be an integral enzyme in lignin biosynthesis [38]. Involvement of shikimate in this pathway was unexpected, yet appears to be a core feature conserved across land plants. HCT is functionally conserved in *Physcomitrella patens* and *Marchantia polymorpha*, and phylogenetic analysis suggests the function emerged in a progenitor of embryophytes [39]. The underlying reason for shikimate’s integral involvement in phenylpropanoid metabolism has not been definitively solved. It has been hypothesized to play a regulatory role, modulating phenylpropanoid flux in response to changes in primary metabolism [40]. However, no transporter facilitating such a connection to the plastidial shikimate pathway has yet been reported, leaving the tantalizing possibility of *de novo* extra-plastidial shikimate biosynthesis.

The steady advance of genetic resources has also lent some support for the Dual Pathway Hypothesis. Analysis of genes encoding the bifunctional dehydroquinate dehydratase/shikimate dehydrogenase (DHD-SDH) in tobacco surprisingly revealed two copies that were highly conserved, with the exception that one copy lacks any apparent N-terminal plastidial transit peptide [41]. Confocal microscopy of GFP-fusion proteins confirmed the predicted cytosolic localization [41]. Difficulty with recombinant expression prevented direct analysis of biochemical function, but the authors did report that overexpression in tobacco leaves caused increased total SDH activity, consistent with conservation of the enzymatic activity [41]. Notably, these results provide a molecular mechanism for aforementioned results that indicated the presence of a cytosolic SDH isoform that was differentially expressed from the plastidial isoform [35]. No analysis has yet been completed of the cytosolic isoform’s physiological role, or the taxonomic distribution of each isoform.

The recent publication of a petunia genome enabled the identification of only a single copy of a chorismate synthase (CS)-encoding gene in that species [42]. Subsequent analysis revealed the presence of both a plastidial transit peptide and putative peroxisomal targeting signals (PTSs) in the primary sequence, and GFP-fusion proteins localized to both organelles. While silencing of the gene was detrimental to plant development, it was not possible to attribute the effect specifically to either localization, and therefore the purpose of targeting the protein to the peroxisome remains unknown. The authors note that the PTSs are present in only some plant CS enzymes reported to date, and thus the peroxisomal localization is unlikely to be universally conserved across plants.

Similar dual localization has recently been observed for DAHPS in Arabidopsis. A high-throughput analysis for protein–protein interactions in *Arabidopsis thaliana* using a cytosolic 14–3-3 protein as bait revealed two of the three DAHPS isoforms encoded in the *A. thaliana* genome as potential interactors [43], though the known plastidial localization of the DAHPSs would be expected to preclude any physiological relevance to this observation. More recently, though, bimolecular fluorescence complementation experiments provided *in vivo* confirmation of the potential for this interaction for one isoform, DAHPS2, with the resulting fluorescent signal evident in the cytosol [44]. The authors postulated that the interaction occurs prior to import of the nascent DAHPS into the plastids and causes retention of the enzyme in the cytosol despite the intact transit peptide. This phenomenon cannot explain the detection of DS-Co, described above, as the catalytic properties and molecular weights of DS-Co and DAHPS2 are incongruent.

**Figure 2:**
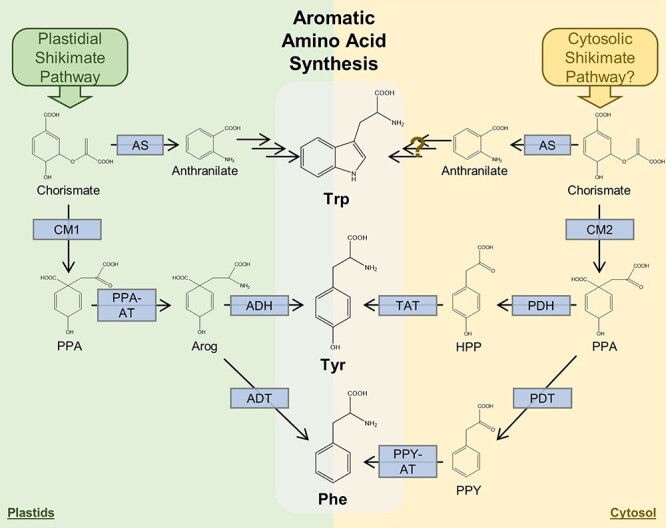
Aromatic amino acid biosynthesis in plants. Green and yellow background indicates plastidial and cytosolic localization, respectively. Existence of enzymatic steps shown in blue boxes is supported by experimental evidence, as described in detail in the text. Stacked arrows represent multiple steps. Brown question mark designates series of enzymes without experimental support. The presence of a cytosolic shikimate pathway remains unconfirmed, but no other source as been demonstrated cytosolic chorismate, which is necessary to sustain cytosolic AAA production. Abbreviations: ADH, arogenate dehydrogenase; ADT, arogenate dehydratase; Arog, arogenate; AS, anthranilate synthase; CM1, chorismate mutase-1 (plastidial); CM2, chorismate mutase-2 (cytosolic); HPP, 4-hydroxyphenylpyruvate; PDT, prephenate dehydratase; PPA, prephenate; PPA-AT, prephenate aminotransferase; PPY, phenylpyruvate; PPY-AT, phenylpyruvate aminotransferase; TAT, tyrosine aminotransferase.

The most comprehensive evidence to date supporting the Dual Pathway Hypothesis revolves around the need for a source of cytosolic chorismate (Fig. 2), the end-product of the shikimate pathway. Although classic evidence supporting the existence of a cytosolic CM in at least some species dates back to the 1980s, as summarized in the previous section, and this includes molecular evidence from Arabidopsis in the 1990s, the advent of the genomic age revealed widespread, and perhaps universal prevalence of CMs lacking plastid transit peptides (CM2s) across angiosperms [45]. A combination of biochemical and reverse genetic experiments in petunia and Arabidopsis demonstrated that CM2 is a highly efficient CM, and that it is necessary to sustain production of Phe-derived specialized metabolites under conditions that required high Phe biosynthetic flux, thereby providing direct experimental evidence for CM2s involvement in cytosolic production of AAAs [46]. The final two steps of this pathway have also now been elucidated. Alternative transcription of a previously characterized ADT was uncovered by 5’-RACE and qRT-PCR analyses, and verified by western blot to be translated in vivo to form an N-terminal truncated protein, which subsequent analyses confirmed to maintain PDT activity and localize to the cytosol [46]. This PDT produces phenylpyruvate that serves as the substrate for a cytosolic PPY-AT that was recently identified [47]. Interestingly, the PPY-AT has a distinct preference for Tyr as the amine donor, thereby providing a link between cytosolic Tyr and Phe levels [47]. It is notable that the order of the final two reactions of this pathway is reversed relative to plastidial Phe-biosynthetic pathway (Fig. 2), thereby following the route that is prevalent among microbes [1].

**Table 1:** Species in which evidence for each step of the hypothesized extraplastidial shikimate pathway have been reported

**Enzymatic Step**	**Biochemical Evidence Only**	**Molecular Evidence**	**References**
DAHPS	mung bean, parsley, potato, carrot, soybean, alfalfa, peas, squash, wheat, rye, *Vitis* hybrid, *Brassica sp*., *Nicotiana silvestris*, *Chlorella sorokiniana*	Arabidopsis	13,14,16,19,23,24,44
DHQS			
DHD		tobacco	41
SDH	pea	tobacco	10,34,35,41
SK			
EPSPS	pea		10
CS		petunia	41
Phe^a^		petunia, Arabidopsis, angiosperms^b^	45–47
Tyr^a^		Soybean, *Medicago truncatula*, Arabidopsis, angiosperms^b^	45,48,51
Trp^a^	tobacco		32

a Refers to any enzyme of the respective post-chorismate AAA biosynthetic pathways

b Sequence analysis across many plant lineages has revealed widespread occurrence of cytosolic CM isoforms, which can contribute to both Phe and Tyr synthesis

Evidence for cytosolic Tyr production is also mounting. Legumes have a non-plastidic, feedback-insensitive PDH [48], which is likely to use CM2-produced cytosolic prephenate to produce 4-hydroxyphenylpyruvic acid. This enzyme is related to plastidial ADHs, but is part of a phylogenically distinct clade that is represented only in a taxonomically restricted group of plants [49]. Reverse genetic analysis is consistent with a bona fide role in cytosolic Tyr production under high flux conditions, though no effect on Tyr levels is observed under standard circumstances [50]. An aminotransferase is necessary to complete this pathway, and while no dedicated hydroxy-phenylpyruvate aminotransferase (HPA-AT) has been specifically identified as being involved, reversible tyrosine aminotransferases (TATs) are present in the cytosol of plant cells [51] and could conceivably work in the Tyr-synthesizing direction if necessary conditions permit it. As no transporters have been reported capable of exporting chorismate from the known site of synthesis in the plastids, a cytosolic shikimate pathway could be the source the necessary substrate for these cytosolic Tyr and Phe biosynthetic pathways.

## Implications of a dual pathway scenario

**Figure 3:**
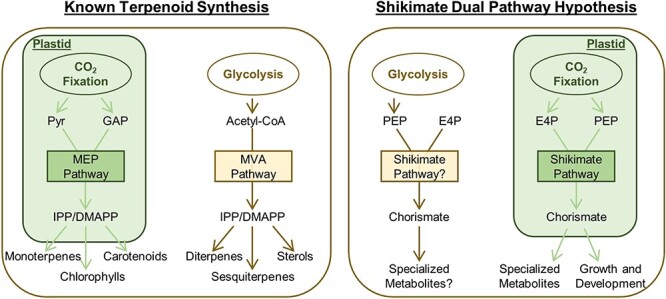
Visualization of parallels between apparent respective redundancy in terpenoid and shikimate pathways. Although they both produce IPP and DMAPP, the MEP and MVA pathways of terpenoid synthesis (left panel) differ in localization, substrate source, and physiological function. A similar scenario is proposed as part of the shikimate Dual Pathway Hypothesis (right panel). For simplicity, intercompartmental transport and metabolic interconnections between central catabolic (glycolysis, pentose phosphate pathway) and anabolic (Calvin-Benson cycle, pentose phosphate pathway, gluconeogenesis) processes are omitted from schematic. Abbreviations not described elsewhere in the manuscript: GAP, glyceraldehyde-3-phosphate; Pyr, pyruvate.

The available data from two different eras support the plausibility of the Dual Pathway Hypothesis from a mechanistic standpoint: there are compartmentally-distinct, functionally-redundant enzymes catalyzing at least some of the intermittent steps between central carbon metabolites and chorismate (Fig. 1) and subsequently to the AAAs (Fig. 2, Table 1). However, this creates new questions around the biological relevance and underlying selective advantage, if any, of such a duplication of the pathway. Although definitive answers to these questions will require more substantial elucidation of the genes involved, the scant existing data in combination with parallels to other redundant pathways—notably, terpenoid biosynthesis—can provide some insight (Fig. 3).

Much like AAA derivatives, terpenoids are a diverse class of thousands of primary and specialized metabolites that are involved in a variety of fundamental and specialized plant functions. As reviewed in [52], their structural cores are synthesized via conjugation of the five-carbon building blocks dimethylallyl pyrophosphate (DMAPP) and its isomer isopentenyl pyrophosphate (IPP). In plants, DMAPP and IPP are readily interconverted, and both can be synthesized by either the plastidial methylerythritol phosphate (MEP) pathway or the extra-plastidial mevalonate (MVA) pathway, the latter of which is primarily cytosolic though some steps localize to the peroxisomes. Despite producing the same end-products, the co-occurrence of these two pathways is ubiquitous across higher plants and common among many photosynthetic ancestors in the plant kingdom.

One notable feature of the duplicated biosynthetic origin of DMAPP and IPP is that each pathway provides precursors for different downstream metabolites. For example, whereas the MEP pathway is necessary for production of monoterpenoids, chlorophylls, and carotenoids, the MVA pathway sustains production of di- and sesquiterpenoids, as well as sterols [52]. The differential localization of downstream enzymes alone cannot explain the evolutionary advantage of this dichotomy, since exchange of IPP across the plastid membrane readily occurs [53]. Thus it is likely that this division of labor instead reflects the benefit of differential regulation [52]. This would not only facilitate altering flux towards one type of compound without modifying the production of others, but can also coordinate downstream metabolite production with availability or alternative utilization of specific pools of upstream precursors (cytosolic acetyl-CoA for the MVA pathway and plastidial pyruvate for the MEP pathway) [52] (Fig. 3).

The same principles of differential regulation of compartmentally-distinct pathways intrinsic to terpenoid metabolism may also underly production of pathway-specific shikimate-derived metabolites. Indeed, biochemical analysis of DS-Mn and DS-Co activities across different conditions are consistent with this. In suspension culture, DS-Mn activity rises quickly then peaks in early exponential phase, corresponding to active primary metabolism and protein synthesis, whereas Ds-Co activity peaks in later exponential phase, more consistent with a role in specialized metabolism [54]. Similar differential changes in activity were observed at different stages of callus differentiation in *Brassica juncea* [55]. In addition, Ds-Mn activity, but not DS-Co activity, increased in parsley with fungal elicitor treatment [23], and a comparable effect was observed in potato upon wounding [56]. In *Vitus* suspension cultures, expression of DS-Co, but not DS-Mn, correlated with anthocyanin accumulation [25], likely altering flux through subsequent steps of any cytosolic pathway.

Differential regulation between compartments is readily apparent in the post-chorismate steps of this metabolic network. Unlike the plastidial CM1, which is subject to strict allosteric regulation, recombinantly-produced cytosolic CM2 appears completely devoid of such regulation [45, 57]. Likewise, the cytosolic PDH, despite homology to the plastidial ADH, lacks sensitivity to allosteric inhibition by Tyr [48]. Results from petunia flowers also suggest differential regulation at the transcriptional level, as CM2 displays a different tissue, developmental, and temporal expression profiles from those of CM1 [46, 57]. It was hypothesized that plastidial biosynthesis requires tighter regulation to maintain narrow AAA homeostasis to support the general needs of plant cells, whereas the cytosolic pathway’s reduced regulation permits rapid production of AAAs to meet transient specialized needs. Indeed, Arabidopsis null CM2 mutants, in which the cytosolic Phe biosynthetic pathway is abolished, have wildtype-like levels of AAAs, suggesting baseline AAA production is unimpeded [46]. However, whereas the wound response of wildtype Arabidopsis involves a burst in production of Phe-derived volatiles, no such response is evident in the mutant lines [46], indicating the cytosolic pathway is necessary to provide Phe substrate for this response.

Response-specific needs of the two pathways may also explain the observation that the plastidial shikimate pathway is subject to redox regulation, with at least two steps, DAHPS and chorismate synthase, being redox sensitive [58, 59]. Since reducing equivalents are produced in the plastids by the light reactions of photosynthesis, and indeed are a critical component of Calvin-Benson Cycle regulation [60], it is likely that this coordinates carbon utilization by the shikimate pathway with carbon fixation and energy production via photosynthesis [61]. This would ensure that growth-related production of AAAs and their derivatives is restricted during periods when insufficient precursors are being produced to sustain other cellular processes. This effect would be compounded with the previously reported sensitivity of the DAHPS reaction rate to substrate pool sizes [62, 63]. Any cytosolic shikimate pathway would not be exposed to the same diurnal redox variation, and thus would be better suited to non-growth related processes, such as stress-response metabolite production, that need not be intrinsically linked to photosynthetic precursor supply. On the other hand, a cytosolic shikimate pathway would be dependent on glycolysis derived PEP, and therefore may require coordination with glycolysis or cytosolic PEP-consuming reactions. Indeed, the reported allosteric sensitivity of PEP carboxylase to shikimate and phenylpropanoid compounds has been proposed to be mechanism for coordinating distribution of PEP between central carbon metabolism and specialized metabolite formation [64, 65], a level of regulation that would take on increased importance if the two pathways were directly competing for the same precursor pool. Such coordination may be of even higher importance in C4 and CAM plants due to their reliance of PEP for photosynthetic function.

In addition to how the different pathway would derive precursors from, and provide substrate for, upstream and downstream metabolic processes, the two pathways would likely differ in their interactions with other co-localized pathways. This has already been observed with a metabolic interaction of the cytosolic Phe pathway with Trp-dependent auxin biosynthesis in which shared intermediates of the pathways create cross-talk [63, 66, 67]. While some such interactions could be beneficial, such as when contributing to metabolic coordination, other interactions may unproductive or even detrimental. For example, *in vitro* activity of all three known plastidial DAHPS isoforms was recently shown to be eliminated in the presence of caffeate [62], a cytosolic intermediate of the phenylpropanoid pathway, but compartmentalization prevents any interference with plastidial shikimate pathway function *in vivo*. As the potential for these interaction varies with alterations in the overall cellular metabolic landscape across conditions, growth state, and cell type, the ability to shift biosynthetic flux of a common product between pathways in separate compartments creates flexibility to avoid situation-specific detrimental inhibition in one organelle or the other.

Finally, the existence of two distinct, spatially separated and differentially regulated pathways creates evolutionary plasticity. Plants have intrinsic adaptive mechanisms that have facilitated evolution of the specialized metabolic diversity necessary for plants to survive and thrive in varied environments [68]. Redundancy of individual enzymes is one such adaptive mechanism [68]. However, redundancy of pathways that contribute to both primary and specialized metabolism could be a powerful adaptive mechanism, as one could be adapted, including dramatic changes to regulation of total flux through the pathway, to specialized needs without interfering with core cellular function. Thus the redundancy of terpenoid biosynthetic pathways, a feature that is unique to—and nearly universal in—the plant kingdom, could underlie the extreme diversity of specialized terpenoids and consequently adaption to varied environments [69], and a similar whole-pathway duplication for chorismate and subsequently AAAs may have facilitated evolution of similar diversity among aromatic specialized metabolites.

## Future perspectives and practical implications of the dual pathway hypothesis

The possibility of a complete second, compartmentally-distinct pathway for production of chorismate and AAAs, as posited by the Dual Pathway Hypothesis, has dramatic implications for future research. First and foremost the hypothesis presents the necessity of identifying and characterizing any genes encoding cytosolic enzymes that catalyze shikimate pathway and AAA biosynthesis steps (Fig. 1), which will allow direct experimental testing of the hypothesis and elucidation of the division of labor between the compartments. As described above, decades-old results provide the foundation for such study, but modern tools and technologies are driving new progress in this area, and will enable assessment of whether compartmentalization of chorismate and AAA biosynthesis is universal across the plant kingdom or specific to certain lineages and/or tissues. This generated knowledge will not only improve our fundamental understanding of natural plant function, but also lay the groundwork for practical improvement of crop production.

Due to the plethora of metabolites derived from, and biological processes dependent on, the shikimate pathway, this pathway and its downstream branches are frequent targets for agricultural enhancement. Previous metabolic engineering strategies, which have almost exclusively targeted the plastidial pathways, have had mixed success in modulating phytochemical production [63, 67]. An active parallel pathway, or even individual steps, presents new targets for engineering, increasing our biotechnological toolbox. Furthermore, due to the interconnected nature of metabolism, any engineering attempt is subject to contextual constraints that limit the expected outcome, as has previously been reported in AAA production [63, 66]. Utilization of plant compartmentalization in biotechnology approaches is a promising method for optimizing metabolic engineering [70]. This would enable tailoring the engineering strategies to the desired conditions, tissues, and species, thereby maximizing production of the desired compounds while simultaneously limiting detrimental impact on the myriad essential processes that are interconnected in one or the other shikimate pathway. However, successful implementation of such strategies will necessitate consideration of all relevant natural catalysts within the different compartments, thus underscoring both the practical and fundamental importance of clarifying the accuracy of the Dual Pathway Hypothesis.
